# Virtual Models Using Augmented Reality May Provide a Suitable Supplement, Although Not a Physical Specimen Replacement, in Pathology Education

**DOI:** 10.1007/s40670-023-01809-9

**Published:** 2023-06-17

**Authors:** Christian Moro, Dianheng Bu, Aditya Gadgil, Gordon Wright, Cindy J. Jones

**Affiliations:** 1 Faculty of Health Sciences and Medicine, Bond University, Gold Coast, 4229 Australia; 2 Menzies Health Institute Queensland, Griffith University, Brisbane, Australia

**Keywords:** Medical education, Technology-enhanced learning, Blended learning, Mixed reality, Disease education, Pathology curriculum, Blended learning

## Abstract

There is a growing trend towards using virtual models within medical programs. In some disciplines, the use of human samples or cadavers is increasingly being replaced by technology-enhanced modes of delivery. Although this transition can occur with some success, the impact of virtual representations to replace depictions of disease states from dissected samples displayed in acrylic pathological specimen jars has never been investigated. This study assessed medical student perceptions of replacing teaching through physical specimens (i.e. specimen jars or real tissue) with virtual models across cardiovascular, neural, musculoskeletal, haematology, endocrine and immunological pathology curricula. Seventy-four year 2 (*n* = 31) and year 5 (*n* = 43) medical students participated in the study. After being provided with a demonstration of a potential tablet-based lesson on lung pathology using augmented reality, participants completed a Likert-scale survey and provided written feedback. Questions requested thoughts on the usefulness of the 3D-virtual model compared to physical specimens and whether current teaching in pathology could be replaced by technology-enhanced practices. Most students (58.15%) disagreed on the replacement of physical specimens with virtual models. Furthermore, over half the students (55.4%) indicated that the replacement of physical specimens with augmented reality models would not be beneficial for pathology learning. Nearly two-thirds of students believed that the absence of physical specimens would negatively impact their knowledge. Nonetheless, many students would appreciate the opportunity to revise pathology away from the labs with virtual options. As such, an overwhelming number of students (89.2%) would prefer having both physical specimens and virtual models for learning. This study identifies that technology-enhanced learning may be a suitable supplement alongside traditional hands-on teaching but should not replace the use of pathological specimens within a medical curriculum.

## Introduction

Virtual three-dimensional (3D) models are increasingly used to facilitate medical education worldwide. Displayed through computer monitors, smartphones, tablets and other digital devices, it is becoming commonplace for medical students to learn a variety of concepts from both real and virtual human body structures [1], as well as real and virtual simulated patients. This type of innovative technology is currently augmenting the way in which educators teach and the methods by which students engage with course content. Across disciplines such as physiology, anatomy, pharmacology and neuroscience [2], 3D models have enabled enhanced learning experiences, as well as a modern way of content delivery for both students and educators. However, although novel technology-enhanced learning is now embedded throughout many other disciplines, the study of pathology remains unique and grounded in realism. As a clinical specialty, some of the core competencies for students to learn in pathology, for example, an understanding of disease mechanisms, integration of mechanisms into organ system pathology and application of pathobiology to diagnostic medicine [3], are not well-translated to study from an entirely virtual environment. There remains a benefit for students to learn from ‘real’ human specimens which may not be effectively replaced by virtual 3D models. The physical specimens also allow provide a stimulus for educators to engage students in conversation on the signs and symptoms that a patient may present with, a differential diagnosis in addition to clinicopathologic features and patient management. Although the current technology does allow for the creation of virtual models in pathology, in many cases these digital representations are often unrealistic or lack the multifarious and complex features of disease presentations provided with pathological real-life samples.

The introduction of virtual models to supplement learning in pathology has not yet been employed widely, with most new resources arising from pathological slide databases or gross pathology photos. Some success has been observed in the creation of a virtual kidney model to teach veterinary [4] or animal pathology [5], although this remains largely in the early-research stages. In other disciplines, such as anatomy and physiology, it is commonplace to teach using digitally created models. This often overlaps slightly with pathology curricula, such as when teaching about stroke [6], dementia [7], brain physiology [8], tumours [9], disorders of the spine [10] and diseases in general [11, 12]. Once these models are created, there are a variety of options to display this in true 3D to the learner. One novel technology of particular interest is augmented reality (AR). In AR, using a camera and screen digital models can be superimposed into the real world. The user is then able to interact with both the real and virtual elements of their surrounding environment [13].

Although there has been a relatively slow uptake towards introducing technology as a core component of pathology teaching, there are areas where digital tools are already providing some benefit to the discipline [14]. For example, recent discussions have surrounded the benefits of using social media and online connectivity [15, 16]. In many cases, this involves developing a community of practice, assisting to educate early-career pathologists. An active online and connected community could assist in patient diagnosis through the sharing of online images or case-specific queries [17, 18]. A growing worldwide virtual community of pathologists is present across Twitter and Facebook, implementing a new way of learning [19, 20]. During the COVID-19 outbreak, there was some success in pathology education being run through e-learning, although this was usually highly structured and guided by an educator, with a two-way interactive experience taking place [21]. There have been acceptable accuracy rates for using digital microscopy compared to glass slides [22, 23], meaning that the migration to a largely virtual curricula across some aspects of pathology in some universities may be unavoidable.

This places pathology in a unique position amongst many of the other disciplines within a medical program. There is potential need for ‘real’ pathological specimens, samples and examples when teaching, which may not be suitably replaced by virtual 3D models. On the other hand, there is a growing benefit in introducing technology to the discipline, although this is usually peripatetic (engaging social media to grow a community of practice). As such, the decision of where to embed technology, how much technology and whether virtual 3D models are suitable in any scenarios must be taken with caution and evidence-based support. The first step to achieving this is to assess this consideration from the learner’s perspective. This study aims to investigate student perspectives on the potential for replacing some of their pathology curricula, commonly learnt with real pathological specimens, with virtual 3D models displayed through AR.

## Methods

### Participants

The study consisted of second and fifth (final) year medical students currently studying at an Australian university’s medical program where pathology teaching was delivered using physical specimens. This was an undergraduate-entry five-year medical program, with the core pathology taught within year two and three of the curricula. Year 2 students were recruited after laboratory sessions and lectures where the study information sheet and consent form were handed out to them by the research team. Year 5 students were recruited during a postgraduate students’ research conference. Augmented reality was not otherwise used within the program. These two cohorts of students were recruited to ascertain if there was any difference in perception between students with and without clinical experience. Year two students had only completed one year of pathology learning with no clinical placement experience, while year 5 student had already completed all their pathology learning and 2 years of clinical placements. Students who wish to partake in the study completed the consent form and handed it back to the research team.

### Data Collection

Consenting students were directed to the pre-setup research area and given a demonstration on an AR model of the lung via a tablet. The model was created in-house by the author using 3ds Max (v2020, Autodesk, San Rafael, USA). Participants were also provided with an explanatory flyer explaining cadaveric lung specimen and virtual lung model. Students were then each given approximately 10 min to explore the AR model of the lung using the tablet followed by the completion of a paper survey. The survey consisted of three sections that evaluated students’ pathology learning experience using physical specimens and AR models as well as their preferred modality for pathology teaching, developed by the team in consultation with a clinical pathologist. The first section contained 7 questions on a 5-point Likert scale (i.e. 1 = strongly disagree to 5 = strongly agree). Questions sought students’ opinions on the replaceability of physical specimens with AR models and asked if they would enhance their pathology learning compared to viewing physical specimens. The final three questions asked about participants’ learning experiences and sought students’ preference for having both AR models and physical specimens in pathology teaching rather than the replacement of physical specimens with AR models. The second section involved participants selecting from a list of body systems (e.g. respiratory system, cardiovascular system) to ascertain whether they believe AR technology would benefit their learning. The final section of the survey offered open-ended questions for additional commentary.

### Data Analysis

Results from the completed surveys were entered and analysed using IBM SPSS Statistics for Windows Version 24.0 (Armonk, NY: IBM Corp.). Besides descriptive statistics, *t*-test, chi-square and multiple responses analyses were conducted to compare between the two groups of students for age and gender as well as their survey responses with a significant alpha level set at *p* < 0.05. For the three open text evaluation question, a narrative synthesis of the responses was undertaken.

### Ethics

Ethics for the study was approved by the Institutional Human Research Ethics Committee. Participants initially read through an explanatory statement and then signed a formal informed consent form prior to participating.

## Results

A total of 74 year 2 (*n* = 31) and year 5 (*n* = 43) medical students participated in the study. *t*-test and chi-square analyses of demographic questions and the seven Likert-scale questions found no statistically significant difference in responses between the two cohorts of students (*p* ≥ 0.05). Therefore, combined results from both cohorts of students are reported (refer to Table 1). Participants were mostly female (*n* = 41) with an average age of 23 (SD = 2.58).

**Table 1:** Evaluation of using physical specimens and AR models in pathology learning and teaching (*n* = 74)

**Questions**	**Strongly disagree**	**Disagree**	**Neutral**	**Agree**	**Strongly agree**
1. Displaying a specimen via AR can replace physical pathology specimens (i.e. ‘pots’) in pathology learning	25.7%	32.4%	20.3%	17.6%	4.1%
2. Using virtual models, at the expense of physical specimens would assist my learning	21.6%	33.8%	21.6%	18.9%	4.1%
3. I would prefer the convenience of learning from AR models at home, instead of having to attending face-to-face sessions to view physical specimens in the pathology museum	13.5%	28.4%	20.3%	25.7%	12.2%
4. I see benefit in having the ability to view more angles, orientations and features in the AR models, compared to the physical specimens	1.4%	8.1%	18.9%	44.6%	27%
5. Access to physical specimens has not assisted me in my pathology learning	43.2%	36.5%	12.2%	4.1%	4.1%
6. The absence of physical specimens during my studies will negatively impact my clinical knowledge	1.4%	17.6%	18.9%	33.8%	28.4%
7. I would prefer to have both, AR and physical specimens, in my future pathology educational resources	1.4%	5.4%	4.1%	20.3%	68.9%

Most students (58.15%) strongly disagreed or disagreed on the replacement of physical specimens with AR models. Furthermore, over half the students (55.4%) indicated that the replacement of physical specimens with AR models will not be beneficial for pathology learning. Similarly, close to two-thirds of the students (66.2%) believed that the absence of physical specimens will negatively impact their clinical knowledge and either disagreed or strongly disagreed (79.7%) that physical specimens have not assisted in their pathology learning. On the other hand, a large proportion of students (71.6%) believe that AR models are beneficial due to the ability to view more angles and orientations than physical specimens. When students were asked if they prefer the convenience of learning from AR models at home instead of attending in-person sessions with physical specimens, results were mixed with relatively similar proportion of students indicating at-home learning (37.9%) and in-person sessions (41.8%). Despite most participants disagreeing with replacing physical specimens with AR models, an overwhelming number of students (89.2%) prefer having both physical specimens and AR models as a learning resource.

### Questions Related to Specific Organ Systems

Generally, students agreed that AR model would be helpful in the learning of organ sets including cardiovascular (86.1%), neural (81.9%) and musculoskeletal systems (77.8%). In contrast, less than half the students believe AR would be helpful for learning pathology in body systems such as haematology (36.1%), endocrine (41.7%) and immunology (31.9%). Interestingly, there is a significantly higher proportion of second year students who believe that AR models can be useful for pathology teaching for the endocrine system (28.6%, 60.0%) and immune system (19.0%, 50.0%) when compared to fifth year students (*p* = 0.01).

### Open-Ended Text Evaluation

A narrative synthesis of the open-ended text evaluation questions revealed three themes which are (a) learning experience; (b) clinical preparedness and (c) practical consideration.

### Learning Experience

Students were concerned that AR models are not an accurate and authentic representation of physical specimens. Students from both cohorts felt that AR models are often “oversimplified” in terms of colour and shape and are not reflective of the actual anatomy that is complex, “multi-layered, with a lot of tissue depth to the structures”. Moreover, AR models are shown on screens, which is difficult to portray the actual “size and proportion” of the specimen and understand “spatial awareness of structures relative to other”.

Furthermore, AR models cannot portray the “variation” in pathological manifestations and often exhibit a “standardized” view of a pathological condition. Therefore, students are unable to “appreciate the differences in anatomy in different cadavers”.

Although students expressed reservations about the realism of AR models, many echoed the benefit that the software being interactive with the ability to physically “manipulate” specimens in different ways such as enlarging and zooming as well as to “add/remove element” or “quickly navigate and strip away layers of structures”. Many highlighted the “3D views” offered by the software to “see structures that are not easily accessible”. This ability to see “deeper structures” meant that the software could also “demonstrate a wider range of pathologies”.

A concern with AR models is the reduction of student engagement and opportunities for peer discussion “the mode of AR discourages teamwork that is needed in the lab to make good doctors”. Furthermore, “distraction with being on a device” inclines students to “find it better learning by seeing real specimens versus interactive 3D model”.

Even though concerns regarding reduced engagement were present, “self-directed” learning was a major benefit seen by many students. Both cohorts of students stated that accessing these specimens through an application meant that the students could access pathologies from “anywhere”. In addition, the software for the use of AR models is readily available and accessible; students thus felt they have more control over learning “at [their] own pace” and could visualize them “outside of class times”.

### Clinical Preparedness

Students from both cohorts expressed concern over their clinical readiness if AR models were to replace physical specimens. Numerous second year students highlighted that the “tactile learning” from feeling and exploring the physical specimens assists in their understanding and memory of the pathology. Without it, they are concerned that this will “impact recognizing pathologies in patients/biopsies” during clinical placements. Furthermore, without the experience of learning with physical specimens during pre-clinical studies, students may not be “comfortable in handling real specimens” during clinical settings. Similarly, year 5 students raised similar concerns that AR models do not provide “knowledge of actual real-life anatomy” and that one “cannot use surgical skills on AR” in clinical settings. Learning through physical specimens helps students to have an “understanding of the different ‘textures’ of the different tissues in the body”, and this “tactile sensation plays an important role in helping to identify pathology during surgery” that is not replaceable with AR models. Participants also commented that they would prefer a “mixture” of physical specimens and AR models, particularly from the year 5 students who felt that AR models could be a “self-study” or revision resource during surgical placements. Some also indicated that AR models could be utilised in assessment.

### Practical Considerations

Timesaving was echoed by many students as they stated that they would also be able to view specimens “outside of the clinical laboratory”. This ability to study specimens in one’s “own time” means students are able to “save time”. The “long-term cost effectiveness” of using AR technology over the cost of cadavers was also highlighted. AR-related application is perceived to be “less expensive to maintain” with cost savings unlike cadavers which require physical infrastructure for storage. Some students indicated potential clinical practice benefits where the AR models can be used to “explain” concepts to “patients”. On the other hand, concerns were also raised regarding the cost of cadavers, as some students commented some “medical schools can’t afford cadavers” which would then result in AR being a benefit on an institution level. One student also stated that AR technology will curtail the potential issue of “cadaver shortage”. Lastly, the “multidimensional” method of learning with AR was also identified as a potential solution to alleviate the “shortage of cadavers”. It should be noted, however, that in this institution, pathologists take part in a coordinated fashion with conventional anatomical dissection and discuss pathological findings from cadavers with students. Participants also expressed reservation that the employment of AR technology as a core teaching component might lead to additional cost required as some students might not have the appropriate technology to utilize AR models. One student commented that “access to latest hardware will inevitably create disparities”. Other concerns identified included difficulties in using and operating AR models, as well as the potential for eye strain after prolonged use.

## Discussion

This study explores the perceptions of using virtual models through AR across medical school pathology learning and teaching. Despite numerous perceived benefits of AR, students ultimately do not see AR as a replacement for preserved specimens but rather an adjunct to pathology learning. Major reservations about AR revolve around its lack of realism and limited ability to present varied pathology. Consequently, students report that they would feel underprepared in real-world settings due to a lack of applied clinical anatomy knowledge and held concerns regarding a decreased confidence in recognizing real pathologies in patients.

Unlike other disciplines covered in the pre-clinical medical curriculum, such as physiology, anatomy and biochemistry, pathology is a unique discipline in its requirement for the clear and concise recognition of diseases in the real world [24]. This means that pathology is a unique area of study that is highly clinically oriented, emphasizing learning with real specimens to understand the three-dimensional image of the various disease processes encountered in clinical practice [25]. This is echoed in our survey, where one of the most significant concerns with AR replacing physical models is the loss of realism, as AR models are not an authentic representation of real pathology. Furthermore, dissection and tissue handling cannot be performed on AR, nor can students learn through tactile sensation from feeling the texture of the specimens [26, 27]. In a study exploring teaching tissue pathology with cadavers, students agreed that working with cadaveric specimens improved their gross tissue identification, handling and dissection techniques which students believe to be highly relevant for their learning [28].

Despite most students believing that AR cannot entirely replace physical specimens, many benefits of AR use have been suggested. AR allows users to isolate and rotate models, which aid in the orientation and visualisation of complex structures. This is reinforced by the use of AR in other medical subjects, especially anatomy, where learning with AR models has shown the potential to improve students’ spatial understanding and 3D comprehension of anatomical structures [29]. AR is also more accessible to students outside regular class and pathology museum times, which enable greater flexibility with self-directed study. Similar points have been highlighted in the use of AR for remote learning which is gaining more importance during the global COVID-19 pandemic [30, 31] or in crammed curricula where there is simply not enough time in the program for scheduling substantial practical laboratory sessions. Students in our study also emphasized that AR can be used in supplementing self-study and exam revision, commensurate with research identifying benefits in its use to supplement learning material in health sciences and medicine [32]. Furthermore, medical museums are considered expensive facilities with high establishment and maintenance outlays [33], which not all medical schools can afford [34]. AR can potentially aid in reducing the discrepancy and improving equity in access to pathology educational resources [35]. This also means that virtual models may have a role in filling-the-gap if institutions do not have adequate pathological samples, large class sizes or access to cadaveric specimens.

### Limitations and Future Research

There were some limitations identified during the study which may have negatively impacted on the results. The first and major limitation was the fact that the study was limited to students from a single university who were recruited after scheduled lectures via convenience sampling. Furthermore, even though the students were shown a demonstration of the AR technology, they were only allowed to see one example of how a virtual model would be presented using a tablet and then were then asked to recall past physical models that they had utilised in class for comparison. This limited period, restriction to lung pathology and lack of recent exposure to physical models meant that students may not have been equally comparing the experiences between delivery modes. Finally, AR technology only offers a single sample that can be viewed by the students. This lack of variety means that the students may not have the same opportunity to see appropriate variations in pathology and therefore may have answered the AR technology unfavourably. Future studies should assess specific measurable outcomes, such as examination performance, to investigate if there is any adverse impact to student knowledge if virtual models are used as a replacement for physical specimens. In addition, assessing perceptions over a linger time period, such as a whole semester, would provide further insights into the use cases for this technology in pathology.

## Conclusion

In the present study, students believe that while AR may be beneficial for studying, physical specimens are still essential to pathology learning. It is no surprise that 89.2% of students preferred a mixture of both modalities for their pathology learning. Therefore, medical programs that choose to shift to AR entirely are likely to receive criticism from students. Currently, the demand for shorter postgraduate courses and greater emphasis on problem-based learning skills has led to less teaching of pathology content [36]. In particular, learning with physical specimens in pathology museums continues to decrease [33]. Consequently, fewer medical students view pathology as useful knowledge for their career [37]. There is some identified need for improvements in the current pathology learning experience. To address this, AR has demonstrated improved enthusiasm in learning in other disciplines and furthermore greater motivation and effectiveness for pathology studying when used together with physical specimens [38, 39].

## Data Availability

The datasets generated during and/or analysed during the current study are available from the corresponding author on reasonable request.
